# Effects of Dietary Protein and Lipid Levels on Growth, Metabolism, Antioxidative Capacity, and Fillet Quality of Adult Triploid Rainbow Trout Farmed in Net Cage

**DOI:** 10.1155/2023/4733343

**Published:** 2023-05-30

**Authors:** Songjing Cao, Lingling Guan, Changzhong Li, Guoliang Sun, Haining Tian, Ruijian Sun, Jun Tu, Yuqiong Meng, Rui Ma

**Affiliations:** ^1^State Key Laboratory of Plateau Ecology and Agriculture, College of Ecological Environmental Engineering, Qinghai University, Xining 810016, China; ^2^Tongwei Agricultural Development Co., LTD., Chengdu 610000, China

## Abstract

The research is aimed at investigating the effects of dietary protein and lipid levels on adult triploid rainbow trout growth performance, feed utilization, digestive and metabolic enzyme activities, antioxidative capacity, and fillet quality. Nine diets containing three dietary protein levels (DP) (300, 350, and 400 g kg^−1^) and three dietary lipid levels (DL) (200, 250, and 300 g kg^−1^) were prepared using a 3 × 3 factorial design. In freshwater cages, 13,500 adult female triploid rainbow trout (3.2 ± 0.1 kg) were cultured for 77 days. Triplicate cages (500 fish per cage) were used as repetitions of each experimental diet. The findings revealed that as DP increased to 400 g kg^−1^ and DL raised to 300 g kg^−1^, the weight gain ratio (WGR) elevated significantly (*P* < 0.05). However, when DP ≥ 350 g kg^−1^, WGR was similar in the DL250 and DL300 groups. As DP raised to 350 g kg^−1^, the feed conversion ratio (FCR) notably decreased (*P* < 0.05). In the DP350DL300 group, lipids had a protein-sparing impact. High DP diet (400 g kg^−1^) generally improved fish health status by increasing antioxidant capacity in the liver and intestine. A high DL diet (300 g kg^−1^) showed no harmful effect on hepatic health based on plasma levels of alanine aminotransferase (ALT) and aspartate aminotransferase (AST) and antioxidant capacity in the liver. For fillet quality, a high DP diet could increase fillet yield, improve fillet hardness, springiness, and water-holding capacity values, and inhibit the production of off-flavors caused by n-6 fatty acids. A high DL diet could increase odor intensity, and EPA, DHA, and n-3 fatty acid concentrations decrease the thrombogenicity index value. The maximum fillet redness value was discovered in the DP400DL300 group. Overall, for adult triploid rainbow trout (≥3 kg), the minimum recommended DP and DL according to growth performance were 400 and 250 g kg^−1^, respectively; DP and DL based on feed utilization were 350 and 200 g kg^−1^, respectively; DP and DL based on fillet quality were 400 and 300 g kg^−1^, respectively.

## 1. Introduction

Rainbow trout (*Oncorhynchus mykiss*), belonging to the order *Salmoniformes* and the family *Salmonidae*, is an essential cold-water aquaculture fish [1]. Triploid rainbow trout are more sterile than diploid fish. They can minimize energy consumption for gonad development, avoid reproductive problems (i.e., high mortality and the decline in fillet quality), and shorten the culturing period. Triploid rainbow trout are particularly attractive commercial fish due to their large size (≥2.5 kg) and great economic worth [2].

A better understanding of nutritional requirements is crucial for feed development and culture technologies of triploid rainbow trout. Previous studies, however, have demonstrated that triploid fish have different nutritional metabolisms and requirements than diploid fish [3]. Therefore, the nutritional requirements of triploid rainbow trout need to be evaluated separately. Concerning practical diet development, appropriate protein, and lipid concentrations in feeds are constitutive elements influencing fish growth [4, 5]. In aquafeeds, protein is the most significant and costly component [6]. Lipid is a nutrient with high bioavailability in salmonids [7], which can more efficiently store protein to use in fish development [8, 9] and decrease feed costs and nitrogenous pollutants in the environment [10, 11]. The protein-sparing effect of lipids has been determined in the literature [12]. In addition, a diet with appropriate protein and lipid contents not only ensures highly efficient use of energy but also improves the nutritional composition of fish and enhances their immunity [13]. As a result, examining the appropriate level of protein and lipid in the diet of farmed fish is beneficial for researching and developing a cost-efficient, nutritionally balanced, and ecologically friendly aquatic feed [14].

Numerous previous studies on the protein and lipid requirements of diploid rainbow trout were mainly focused on the juvenile and subadult stages by evaluating fish growth [15, 16], digestion and absorption [17], metabolism [18], fish health [18], and fillet quality [19, 20]. Nevertheless, to the best of our knowledge, there has been relatively few research focusing on large-size (≥2.5 kg) rainbow trout.

Regarding triploid rainbow trout, our earlier research has indicated that the minimum requirement of lipid and protein in fish (initial weight: 233 g) was 233 g kg^−1^ [3] and 458 g kg^−1^ [21], respectively. Based on fish growth, the recommended level of lipid and protein for fish (initial weight: 1.5 kg) was 250 g kg^−1^ and 400 g kg^−1^, respectively (unpublished data). As a result, this study was conducted using a two-factor design to explore the recommended level of lipid and protein for adult triploid rainbow trout (≥3 kg) based on growth performance, feed utilization, digestion and metabolism, antioxidant response, and fillet quality. It is expected to provide a basis for the establishment of a nutritional database and the development of an efficient and environmentally diet of triploid rainbow trout.

## 2. Materials and Methods

### 2.1. Experimental Diets and Feeding Trial

Based on the results of our previous studies, the minimum level of lipid and protein in the diet for triploid rainbow trout (initial weight: 1.5 ± 0.1 kg) was 250 and 400 g kg^−1^, respectively, and the recommended level of protein in diets was 360 g kg^−1^ for rainbow trout (>1,500 g) [22]. A two-factor (3 × 3) experiment designed with three dietary lipid levels (DL) (200, 250, and 300 g kg^−1^) and three protein levels (DP) (300, 350, and 400 g kg^−1^) was utilized to formulate a total of nine diets labelled DP300DL200, DP300DL250, DP300DL300, DP350DL200, DP350DL250, DP350DL300, DP400DL200, DP400DL250, and DP400DL300, respectively. The major lipid and protein sources were fish oil and fish meal, respectively.  Table 1 provides an overview of the experimental diets' formulations as well as their approximate compositions. Tongwei Agricultural Development Co., LTD. in China was responsible for producing all of the experimental diets with 7 mm expanded feed. Feed processing and storage were carried out in the manner previously described [21].

A total of 13,500 adult triploid rainbow trout (female) weighting 3.2 kg were taken from the local fisheries in Qinghai province of China. These trout were then randomly dispersed into 27 net cages of 8 × 8 × 8 meters, with 500 fish in each cage. During the feeding trial, three replicate net cages (*n* = 3), which were placed in a reservoir in the Qinghai province of China, were chosen to culture the trout fed one of the nine different experimental diets. Fish were hand-fed at 08 : 30 and 16 : 30 using the standard of apparent satiation (stop feeding when most fish were not actively eating). The number and weight of dead fish and feed consumption were documented throughout this period. The feeding trial of 77 days was carried out in the autumn (from October to December). Dissolved oxygen (7.4 ± 1.6 mg L^−1^) and water temperature (11.1 ± 3.7°C) were measured daily.

### 2.2. Sample Collection

After 24 h fasting of all the experimental fish following the feeding experiment, all of the fish in each net cage were gathered, and thirty fish were taken at random and weighted following anesthesia with eugenol at a ratio of one to ten thousand (Shanghai Reagent Corp., China). The aforementioned weighting technique was repeated three times, and the mean value of three weights of 30 fish was used as the average final weight of fish in each cage. One ungutted fish per net cage was chosen at random to analyze the whole body composition. Three fish per net cage were chosen to measure body weight and body length after anesthesia, respectively, and then blood samples were obtained from the caudal vein of each fish with 5 mL syringes. Plasma samples were separated by centrifuging at 4,000 × g for 10 min and combined to form one biological duplicate with three fish from one cage of comparable volume. Three bled fish were then dissected to obtain viscera, abdominal fat, hepatic, intestine, and two fillets. In order to determine the viscerosomatic index (VSI), relative intestinal length (RIL), fillet yield (FY), abdominal fat index (AFI), and hepatosomatic index (HSI), the weights of each fish's viscera, abdominal fat, liver, and fillet, as well as the length of the intestine were measured. Liver and intestinal samples were combined to form one biological duplicate with three fish from one cage of comparable size. Three biological replicates (*n* = 3) were created for each diet group, and all samples were frozen in liquid nitrogen and subsequently kept at -80°C for further analysis. The fillet samples were prepared on the basis of the method described in our previous study [23].

### 2.3. Biochemical Analysis

#### 2.3.1. Analysis of the Approximate Composition of Experimental Diets

The Association of Official Analytical Chemists' standard procedures were used to assess feed ingredients and experimental diets for dry matter, crude protein, and crude lipid [21]. The samples were burned in an adiabatic bomb calorimeter (IKA Calorimeter, C400, Germany) to analyze the total quantity of gross energy in diets.

#### 2.3.2. Analysis of Body and Tissue Compositions

To assess moisture content, whole body, liver, and fillet samples were freeze-dried to a consistent weight at -45°C. The nitrogen content (*N* × 6.25) was measured using a Dumas Nitrogen Analyzer (Dumatec™ 8000, FOSS, Denmark). The results were used to calculate the crude protein contents in the whole body, liver, and fillet. The crude lipid contents in the whole body, liver, and fillet were extracted using the Folch et al. [24] method of chloroform: methanol (2 : 1, *v*/*v*). A commercial test kit with the anthrone-sulfuric acid colorimetric method (Nanjing Jiancheng Bioengineering Institute, China) was used to determine the glycogen content in the liver.

#### 2.3.3. Analysis of Enzyme Activity and Plasma Biochemistry Parameters

Following the procedure described by Ma et al. [21], the homogenization of liver and intestinal tissue samples was performed using an electric homogenizer (XHF-D, Xinzhi, China). Total protease and lipase activities in the intestine were measured using the procedures reported by Meng et al. [3]. Total antioxidative capacity (T-AOC, by ferric ion reducing antioxidant power method), protein carbonyl (PC, by ultraviolet colorimetric method), and malonaldehyde (MDA, by thibabituric acid method) levels in the plasma, intestine, and liver were analyzed using commercial kits. Lipoprotein lipase (LPL), hepatic lipase (HL), and total lipase (by colorimetric method), as well as ALT, AST (by Reitman Frankel's method), and glutamate dehydrogenase (GDH, by nicotinamide adenine dinucleotide rate method) activities in the liver, were estimated using commercial kits. The tissue protein content was evaluated using a commercial kit and the Coomassie brilliant blue technique. All analyses were carried out on three biological duplicates of each group.

Additionally, plasma ALT and AST levels were measured using established clinical procedures in an automated biochemistry analyzer (ADVIA 2400, SIEMENS, Germany) in a recognized hospital.

#### 2.3.4. Analysis of Fillet Color

The color card (SalmoFan™, DSM, Netherlands) was used to analyze the redness of the fillet visually. Fillet color described by lightness (*L*^∗^), redness (*a*^∗^), yellowness (*b*^∗^), chroma (*C*^∗^_ab_), and hue (*H*_ab_°) was also assessed directly using a colorimeter (CR-400, Minolta, Japan) on the basis of our previous study [23].

#### 2.3.5. Analysis of Fillet Texture

Fillet pH and water holding capacity (WHC) was assayed by the methods of Meng et al. [23] by using a solid electrode (Inlab® Solids Pro-ISM, Mettler Toledo, Swiss) and a texture analyzer (TMS-PRO, FTC, USA), respectively.

Texture profile analyses (TPA) were performed on the texture of fillets using a texture analyzer (TMS-PRO, FTC, USA) following the methodology given in our earlier work [25]. The instrumental definitions are defined as follows:

Fracture (*N*): the first significant break in the first compression cycle;

Hardness (*N*): peak force in the first compression cycle;

Adhesiveness (mJ): the negative area for the first bite;

Cohesiveness: positive area of the second compression cycle/positive area of the first compression cycle;

Springiness (mm): the end of the first bite height—the start of the second height;

Chewiness (mJ): hardness × cohesiveness × springiness.

#### 2.3.6. Analysis of Fillet Fatty Acid Composition

The fatty acid analysis was performed on the collected lipid following the fillet lipid assay. The fatty acids were methyl esterified and analyzed using gas chromatography-mass spectrometry (GC-MS, QP2020, Shimadzu, Japan), and nutritional value indicators of fatty acids (i.e., atherogenicity index (AI) and thrombogenicity index (TI)) were calculated following the methodology given in our earlier work [26].

#### 2.3.7. Analysis of Fillet Odor

The approach reported by Ma et al. [27] was utilized to evaluate the volatile compounds in the fillet using gas chromatography mass spectrometry (GC-MS, QP2020, Shimadzu, Japan) coupled with automated solid-phase microextraction (SPME) equipment. Odor-active compounds were utilized to assess the effect of diet group on fish fillet odor (odor activity value (OAV) ≥ 1).

### 2.4. Calculations

(1) $$\begin{array}{c} \text{Survival }\left(\%\right)=100\times \frac{\text{FN}}{\text{IN}},\\ \text{Weight gain rate }\left(\text{WGR},\%\right)=100\times \frac{\left[\text{FBW }\left(\text{g}\right)-\text{IBW }\left(\text{g}\right)\right]}{\text{IBW }\left(\text{g}\right)},\\ \text{Feed conversion ratio }\left(\text{FCR}\right)=\frac{\text{FW }\left(\text{g}\right)}{\left[\text{FBW }\left(\text{g}\right)-\text{IBW }\left(\text{g}\right)\right]},\\ \text{Feed intake }\left(\text{FI},\%\;{\text{day}}^{-1}\right)=100\times \frac{\text{FW}\left(\text{g}\right)}{\left[\text{days}\times \left(\text{IBW }\left(\text{g}\right)+\text{FBW }\left(\text{g}\right)/2\right.\right]},\\ \text{Condition factor }\left(\text{CF}\right)=100\times \frac{\left[\text{BW }\left(\text{g}\right)\right]}{{\left[\text{BL }\left(\text{cm}\right)\right]}^{3}},\\ \text{Viscerosomatic index}\left(\text{VSI},\%\right)=100\times \frac{\text{VW }\left(\text{g}\right)}{\text{BW }\left(\text{g}\right)},\\ \text{Abdominal fat index }\left(\text{AFI}\right)=\frac{\text{AFW }\left(\text{g}\right)}{\text{BW }\left(\text{g}\right)},\\ \text{Hepatosomatic index }\left(\text{HSI},\%\right)=100\times \frac{\text{HW}\left(\text{g}\right)}{\text{BW}\left(\text{g}\right)},\\ \text{Relative intestine length }\left(\text{RIL}\right)=\frac{\text{IL }\left(\text{cm}\right)}{\text{BL }\left(\text{cm}\right)},\\ \text{Fillet yield }\left(\text{FY},\%\right)=100\times \frac{\text{MW}\left(\text{g}\right)}{\text{BW}\left(\text{g}\right)},\\ \text{The ratio of protein to energy in diet }\left(\text{mg protein }{\text{KJ}}^{-1}\right)=\frac{\text{crude protein of diet }\left(\text{mg }{\text{g}}^{-1}\right)}{\left[\text{gross energy of diet }\left(\text{KJ}{\text{g}}^{-1}\right)\right]}, \end{array}$$

where FN, IN, BW, FBW, IBW, FW, BL, VW, AFW, HW, IL, and MW represent the final number of fish, the initial number of fish, body weight, final body weight, initial body weight, feed fed weight, body length, viscera weight, abdominal fat weight, hepatic weight, intestinal length, and fillet weight, respectively.

### 2.5. Statistical Analysis

A two-factorial design (3 × 3) was used to assess the interaction between DP and DL. Factorial (two-way) and one-way ANOVA (analysis of variance) were conducted for all the data using SPSS 26.0 software following the approach described by Xu et al. [28]. The data were presented as mean ± standard error.

## 3. Results

### 3.1. Growth Performance and Feed Utilization

Data on growth performance and feed utilization of triploid rainbow trout are presented in Table 2. The interaction (DP × DL), DP, and DL fail to affect FI and survival of the fish (*P* > 0.05). According to two-way ANOVA, DP × DL did not affect FW, WGR, and FCR of triploid rainbow trout (*P* > 0.05). DP significantly affected WGR and FCR (*P* < 0.05). WGR elevated considerably with DP increasing and peaked in the DP400 group, while FCR declined as DP increased to 350 g kg^−1^, then plateaued (350 to 400 g kg^−1^). DL affects FW and WGR significantly (*P* < 0.05), but no effect on FCR was observed. FW and WGR elevated considerably as DL increased; the highest value was found in the DL300 group (*P* < 0.05). When DP ≥ 350 g kg^−1^, the indicators were similar in the DL250 and DL300 groups.

### 3.2. Body Indices and Compositions

Based on two-way ANOVA, the CF value was not affected by DP, DL, and DP × DL (*P* > 0.05, Table 3). DP and DL showed a significant (*P* < 0.05) influence on FY, AFI, and VSI values, while DP × DL had no effect. VSI and AFI values increased and then decreased (*P* < 0.05) as DP increased, and the highest values were found in the DP350 group. The DP400 group showed significantly (*P* < 0.05) higher FY value than the other groups. With DL increasing, the VSI and AFI values raised and peaked in the DL300 group (*P* < 0.05). The FY value in the DL300 group was substantially lower than that in the other DL group. HSI and RIL values were significantly (*P* < 0.05) affected by the DP × DL. The highest HSI value was found in the DL300 group when DP ≤ 350 g kg^−1^, but it was not varied as DL when DP was 400 g kg^−1^. The DP300DL200 group had the lowest RIL value, while the DP350DL200 group had the highest value.

**Figure 1:**
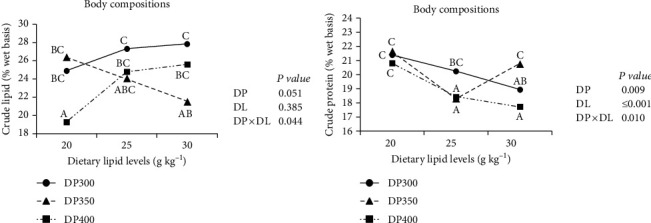
Means of the crude lipid (a) and protein (b) contents of fish body compositions in different diet groups. Values denoted with different letters (A, B,…based on one-way ANOVA) represent that the significant difference is discovered in the interaction (*P* < 0.05).

DP × DL had a significant effect on whole-body crude protein and lipid contents (*P* < 0.05, Figure 1). Whole-body crude lipid content (Figure 1(a)) raised as DL increased and peaked in the DL250 group when DP was 300 or 400 g kg^−1^. However, it decreased with DL increasing when DP was 350 g kg^−1^. Except for the DP350DL300 group, which had high whole-body protein content but low lipid content, whole-body crude protein content declined with DL increasing (Figure 1(b)).

Data on fish liver compositions are presented in Table 4. DP × DL had no effect (*P* > 0.05) on hepatic glycogen, protein, or lipid content. DP significantly affected hepatic lipid, protein, and glycogen contents (*P* < 0.05). The DP400 group had greater protein and lipid contents but lower glycogen content than the DP300 and DP350 groups (*P* < 0.05). DL only affected hepatic glycogen content. The DL300 group had significantly greater glycogen content than the DL200 and DL250 groups (*P* < 0.05).

### 3.3. Digestive Enzyme Activities

**Figure 2:**
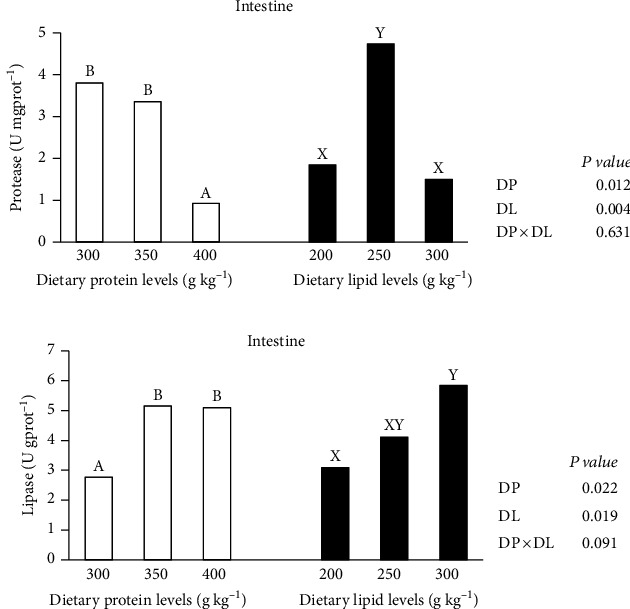
Means of the intestinal activities of protease (a) and lipase (b) in different diet groups. Values denoted with different letters (A, B, and C based on two-way ANOVA) represent that the significant difference is discovered in the dietary protein level (*P* < 0.05) and those with different letters (X, Y, and Z based on two-way ANOVA) represent that the significant difference is discovered in the dietary lipid level (*P* < 0.05).

Data on protease and lipase activities in the digestive tract are shown in Figure 2. The activities of protease and lipase in the intestine were significantly affected by DP and DL (*P* < 0.05), but the effect of DP × DL was not significant (*P* > 0.05). The DP400 group had significantly (*P* < 0.05) lower protease activity than DP300 and DP350 groups. The DL250 group had significantly greater protease activity than the DL200 and DL300 groups (Figure 2(a)). Lipase activity potentiated considerably (*P* < 0.05) with DL rising and peaking in the DL300 group. It also potentiated significantly with DP increasing and peaked in the DP350 group (Figure 2(b)).

### 3.4. Metabolic Enzyme Activities

As seen in Table 5, DP, DL, and DP × DL had no effect on plasma ALT and AST levels. According to two-way ANOVA, the activities of amino acid metabolism and lipid metabolism enzymes in the liver were affected by DP × DL (*P* < 0.05) (Table 5). When DL was 300 g kg^−1^, the DP300 group had notably higher hepatic GDH, ALT, and AST activities than the other groups (*P* < 0.05). Furthermore, ALT and AST activities in the liver showed an increasing trend with DL increasing when DP ≤ 350 g kg^−1^, but the tendency was reversed when DP was 400 g kg^−1^. When DL was 200 g kg^−1^, the DP300 group had the greatest activities of HL and total lipase in the liver, whereas the DP400 group had the highest LPL activity. In addition, when DL was 300 g kg^−1^, the maximum LPL and total lipase activities in the liver were found in the DP350 group.

### 3.5. Antioxidative Capacity

**Figure 3:**
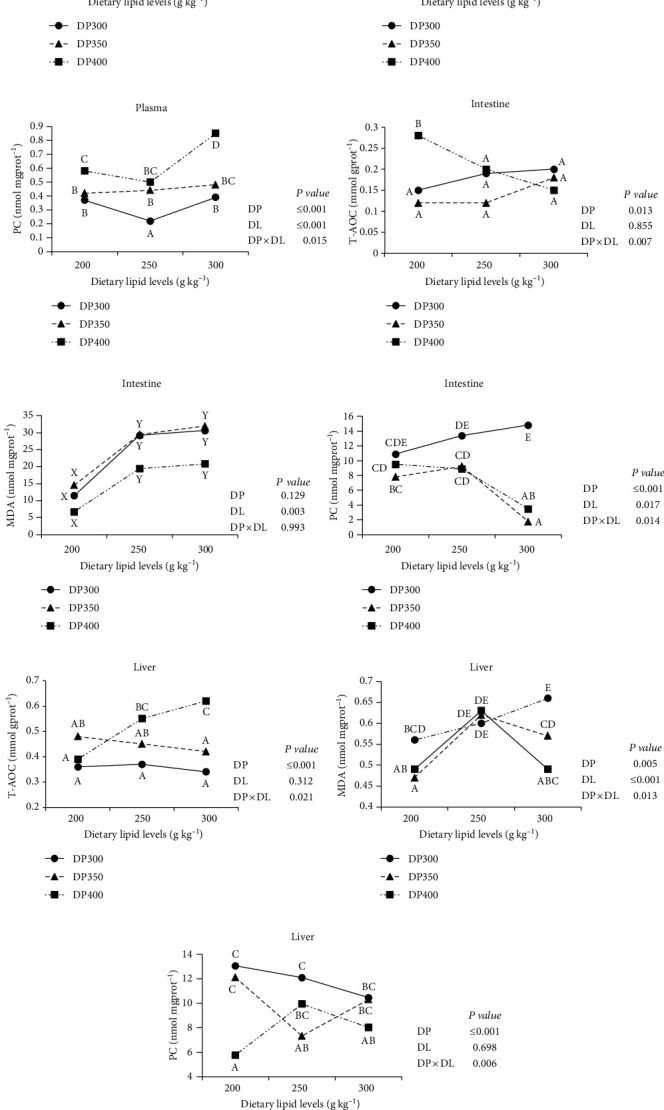
Means of total antioxidative capacity (T-AOC), malondialdehyde (MDA), and protein carbonyl (PC) levels of adult triploid rainbow trout in different diet groups. Values denoted with different letters (A, B,…based on one-way ANOVA) represent that the significant difference is discovered in the interaction (*P* < 0.05), those with different letters (A, B, and C based on two-way ANOVA) represent that the significant difference is discovered in the dietary protein level (*P* < 0.05), and those with different letters (X, Y, and Z based on two-way ANOVA) represent that the significant difference is discovered in the dietary lipid level (*P* < 0.05).

Data on fish antioxidant capacity are shown in Figure 3. DP × DL significantly affected T-AOC level in the plasma, intestine, and liver (*P* < 0.05). When DP was 300 and 400 g kg^−1^, the DL250 and DL300 groups exhibited low plasma T-AOC level (Figure 3(a)). The highest T-AOC level in the intestine was found in the DP400DL200 group (Figure 3(d)). The T-AOC level in the liver enhanced with DL increasing when DP was 400 g kg^−1^, but it did not alter with DL when DP was ≤350 g kg^−1^ (Figure 3(g)).

There was a significant effect of DP × DL on plasma and hepatic MDA contents, but no effect on intestinal MDA content. The DP300DL300 group had the greatest MDA content in the plasma and liver (*P* < 0.05) (Figures 3(b) and 3(h)). In addition, whether DL was 200 or 300 g kg^−1^, the DP400 group showed lower hepatic MDA content (Figure 3(h)). The MDA content in the intestine raised considerably DL increasing and peaked in the DL250 group, but DP had no effect on it (Figure 3(e)).

DP × DL significantly affected the PC contents in the plasma, intestine, and liver. The DP400 group exhibited greater plasma PC content than DP300 and DP350 groups, especially the highest PC content was found in the DP400DL300 group (Figure 3(c)). In the DL300 group, the diets with DP ≥350 g kg^−1^ could reduce PC content in the intestine (Figure 3(f)). When DL was 200 or 300 g kg^−1^, the DP400 group had the lowest PC content in the liver (Figure 3(i)).

### 3.6. Fillet Quality Color

DP, DL, and DP × DL fail to affect fillet redness value assessed visually, *L*^∗^ and *H*_ab_° values (*P* > 0.05; Table 6). DP had a notable effect on the *b*^∗^ value, and the DP350 group had a lower value than the other groups (*P* < 0.05). DP × DL significantly affected *a*^∗^ and *C*^∗^_*ab*_ values. The *a*^∗^ value notably elevated with DL increasing when DP was 400 g kg^−1^, but it reduced with DL increasing when DP ≤ 350 g kg^−1^ (*P* < 0.05). The highest *C*^∗^_ab_ value was found in the DP400DL250 and DP400DL300 groups.

### 3.7. Fillet Quality Texture

Data on fillet texture are presented in Table 7. DP, DL, and DP × DL fail to affect cohesiveness and pH values (*P* > 0.05). DP or DL had the significant influence on fracture, hardness, and adhesiveness values (*P* < 0.05), while the effect of DP × DL was not significant (*P* > 0.05). The DL200 group had the greatest fracture, hardness, and adhesiveness values. The values of hardness and adhesiveness enhanced with DP increasing up to 350 g kg^−1^, and then decreased. DP × DL notably (*P* < 0.05) affected springiness, chewiness, and WHC values. The DP350 group had a lower springiness value than the other groups. The highest springiness and chewiness values were found in the DP300DL200 group. The DP300 group had a lower WHC value than the DP350 and DP400 groups, and the lowest value was found in the DP300DL300 group.

### 3.8. Fillet Quality Odor

Data on volatile compounds with OAVs in the fillet are shown in Table 8. Twenty odor-active compounds were found in the study, including 2 alcohols (1-heptanol and 1-octen-3-ol), 3 ketones (2,3-pentanedione, 2,3-octanedione, and 3,5-octadien-2-one), 14 aldehydes (hexanal, heptanal, octanal, nonanal, decanal, undecanal, (*E*)-2-hexenal, (*E*)-2-heptenal, (*E*)-2-octenal, (*E*)-2-nonenal, (*E*)-2-decenal, (*E, E*)-2,4-heptadienal, (*E, Z*)-2,6-nonadienal, and (*E, E*)-2,4-nonadienal), and 1 furan (2-ethyl-furan). DP, DL, and DP × DL fail to affect the concentrations of 2,3-octanedione, heptanal, nonanal, (*E*)-2-nonenal, (*E*)-2-decenal, and (*E, E*)-2,4-nonadienal. The concentrations of 1-heptanol, decanal, undecanal, (*E, Z*)-2,6-nonadienal, and subtotal concentration of n-9 fatty acids (n-9 derived) increased as DP increasing (*P* < 0.05) and peaked in the DP350 group, while the concentrations of 1-octen-3-ol, 2,3-pentanedione, hexanal, octanal, (*E*)-2-hexenal, (*E*)-2-heptenal, and total concentration (TC) and n-6 derived tended to increase as DP increasing up to 350 g kg^−1^, and then decrease. (*E*)-2-hexenal and (*E, E*)-2,4-heptadienal concentrations increased with DL increasing. The concentrations of 1-octen-3-ol, hexanal, (*E*)-2-octenal, (*E, Z*)-2,6-nonadienal, 2-ethyl-furan, TC, n-3 derived, and n-6 derived notably increased as DL increasing (*P* < 0.05) and peaked in the DL250 group. DP × DL had a significant influence on 3,5-octadien-2-one concentration (*P* < 0.05), it enhanced as DL increasing when DP ≥ 350 g kg^−1^.

### 3.9. Fillet Quality Nutrition

Data on fillet protein and lipid content are shown in Table 4. Fillet lipid content was unaffected (*P* > 0.05) by DP × DL, DP, and DL. Fillet protein content was only affected by DP. The DP300 group had considerably lower fillet protein content than the DP350 and DP400 groups (*P* < 0.05).

Data on fatty acid compositions are shown in Table 9. The contents of C18:2n-6, C18:3n-3, C20:4n-6, total fatty acids (TFA), saturated fatty acids (SFA), monounsaturated fatty acids (MUFA), polyunsaturated fatty acids (PUFA), n-6 fatty acids (∑n-6) contents, and AI value were neither affected by DP × DL nor DP and DL. The contents of C20:5n-3 (EPA), C22:6n-3 (DHA), n-3 fatty acids (∑n-3), and TI value were unaffected (*P* > 0.05) by DP × DL and DP, but were notably affected by DL (*P* < 0.05). The DL300 group had higher EPA, DHA, and ∑n-3 contents than the other groups, while the TI value showed the opposite trend (*P* < 0.05).

## 4. Discussion

The current study discovered that the growth of triploid rainbow trout was enhanced when the fish were fed the diet with DL ≥ 250 g kg^−1^, and there was no negative effect of the high DL diet (300 g kg^−1^) on fish growth. This is substantiated by Meng et al. [3] who pointed out that triploid rainbow trout (initial weight: 233 g) could use high lipid level with no negative effect on fish growth. Besides, the DP400 group in the study had the best WGR. The result was higher than recommended protein level (360 g kg^−1^) of rainbow trout (>1,500 g) [22]. Triploid rainbow trout had higher protein requirements than diploid rainbow trout was also found in our previous study [21]. Overall, the minimum recommended DP and DL for adult triploid rainbow trout (≥3 kg) on the basis of growth was 400 and 250 g kg^−1^, respectively.

Fish growth is also affected by feed utilization; hence, FI and FCR were thoroughly evaluated. DP and DL had no effect on FI in the present study. When DL ≥ 195 g kg^−1^, no effect of DL on FI was also found in subadult triploid rainbow trout [3]. However, Ma et al. [21] discovered that raising DP from 316 to 407 g kg^−1^ improved FI in subadult triploid rainbow trout. The phenomenon was not observed in adult fish, which indicated that large fish fed actively and were not picky eaters. The minimum recommended DP and DL for adult triploid rainbow trout (≥3 kg) based on FCR results was 350 and 200 g kg^−1^, respectively. Fish feed utilization was related to the processes of nutrient digestion. Digestion is performed by a variety of substrate-specific digestive enzymes, and there is a limit to the digestion and absorption of protein and lipid in diet by fish [29]. The current study examined the specific activities of protease and lipase in the intestine and discovered that the DP350 group might potentially improve fish digestion. It might explain why the minimum recommended DP was 350, not 400 g kg^−1^ on the basis of FCR in the study. In addition, the DL300 group enhanced lipase activity but inhibited protease activity in the present study. Similar findings were reported in subadult fish [3]. This might explain why a high DL diet has no effect on FCR.

Based on whole body composition in the study, the growth improvement by the diets with DL ≥ 250 g kg^−1^ was attributed to lipid deposition. Similar findings have been found in other fish species [30]. It was also supported by VSI and AFI values and lipase activity in the study. Meng et al. [31] showed that viscera was the most crucial site of lipid deposition in triploid rainbow trout. The highest VSI and AFI values were found in the DL300 group in the study. In addition, lipase activity potentiated as DL increased, which was similar to the finding in subadult rainbow trout [3]. Combined with the results, triploid rainbow trout in the whole life might have a strong capacity for lipid digestion. The DP350DL300 group had high protein and low lipid contents in the whole body, which was an intriguing finding in the study. It might be related to the protein-sparing effect of lipid in relation to energy generation resulting in the increasement of protein accretion in the fish's body [32].

The liver is a crucial organ in fish, performing metabolic and detoxifying tasks [18, 33]. Liver health is vital to fish health. Our previous study showed that increasing DL lowered the HSI value in subadult rainbow trout [31]. On the contrary, the DL300 group generally had a high HSI value in the present study. The liver is thought to be a key location of lipid and glycogen accumulation in fish [34, 35]. Liver tissue compositions were then assayed, and results showed that hepatic glycogen not lipid content was accumulated in the DL300 group. However, the phenomenon was not observed in subadult triploid rainbow trout [31]. The findings revealed that adult rainbow trout might prefer to use lipid rather than glucose when fed the high DL diet (300 g kg^−1^), which led to more glucose being stored as glycogen. The main reason will require further study. An intriguing finding in the study was that high a DP diet (400 g kg^−1^) might mitigate the increase in HSI value produced by a high DL diet. It was related to decreasing hepatic glycogen content. As seen in earlier studies [36, 37], HSI and hepatic glycogen content were positively correlated with DL and negatively correlated with DP. According to Ma et al. [21], a high HSI value was associated with oxidative damage. Then antioxidative indicators were assayed in the study. T-AOC indicates overall antioxidant capacity, whereas MDA is a key indicator of oxidative damage [38]. In addition, free radicals generated during lipid peroxidation can also denature proteins, and PC is the main indicator of oxidative protein damage [33]. In the study, a high DP diet generally increased hepatic T-AOC level and decreased hepatic MDA and PC contents, especially when DL was 300 g kg^−1^. It was agreed that the optimal DP may enhance antioxidative capacity and benefit fish health [21]. The experimental diets fail to affect plasma ALT and AST levels, which are biomarkers for liver damage [39]. It indicated that liver damage was seemingly absent in the low DP group (300 g kg^−1^) or high DL group (300 g kg^−1^), which were different from the results in subadult fish [3, 21]. Adult fish might have a greater tolerance for diet than subadult fish. The gut is intimately linked to fish digestion, absorption, and immune response [40, 41]. Similar to the results in the liver, the DP400 group generally increased T-AOC level and decreased MDA and PC contents in the intestine. Based on the aforementioned results, a high DP diet was beneficial to fish health, and a high DL diet showed no influence on hepatic health.

Fillet is the most important product produced by salmonids, which is the majority of the edible component for consumers [42]. Thus, the present study examined the impact of DP and DL on the fillet quality of adult triploid rainbow trout. FY was considered an important quality indicator in fillet processing. In the study, the FY value was high in the DP400 group but low in the DL300 group. The variation was opposite with VSI value and might be related to the distribution of nutrients in various tissue. The color of the flesh is extremely important in consumer acceptance of salmonid quality [43]. The DP400DL300 group showed high quality in fillet color because it had the greatest redness value (*a*^∗^). Generally, a firm texture is preferred by consumers of salmon [44]. Previous studies in rainbow trout also found a high DL diet could decrease fillet hardness value [20]. The DL200 group had the greatest fillet hardness, springiness, and chewiness. Nonetheless, fish fed a low DL diet also had the highest fillet fracture value which indicated that the increasement in fragility could result in the decline of fillet quality. For the effect of DP, the DP400 group had higher fillet hardness, springiness, and WHC values, which indicated that high DP is suitable for texture.

In addition to color and texture, the odor of fish flesh is a significant quality measure [45]. The OAV indicates the contribution of each volatile component to the overall odor profile of the fish sample [46]. 20 odor-active compounds may contribute to the overall odor, including 2 alcohols, 3 ketones, 14 aldehydes, and 1 furan compound, according to the OAV (≥1). In comparison to our previous studies on 4 kg triploid rainbow trout fillets [27], most odor-active compound species were the same except pentanal (not detected in the study), 2-pentyl-furan (not detected in the study), and 3,5-octadien-2-one (not detected in our previous study). The oxidative degradation of lipids (particularly unsaturated fatty acids) was the primary source of volatile organic compounds such as alcohols, ketones, and aldehydes [47–49]. Based on previous studies, pentanal and 2-pentyl-furan are mainly derived from n-6 fatty acids [47, 50], but 3,5-octadien-2-one is derived from n-3 fatty acids [51]. Hence, the difference in odor-active compound species was related to the feed composition of the diets which mainly consisted of fish meal and fish oil in the study. The odor description of volatile compounds derived from n-3 fatty acids, such as 3,5-octadien-2-one and (*E*)-2-hexenal, is generally pleasant, while the volatile compounds derived from n-6 fatty acids, such as 1-octen-3-ol and hexanal, may cause off-flavors [49]. The concentrations of n-3 derived and n-6 derived were all enhanced when DL ≥ 250 g kg^−1^ in the study. The results indicated that a diet with DL ≥ 250 g kg^−1^ could increase odor intensity overall, regardless of good or bad. The study discovered an intriguing result that the concentrations of n-6 derived and n-9 derived were enhanced with DP increasing up to 350 g kg^−1^, while n-6 derived dropped as DP increased from 350 to 400 g kg^−1^. It indicated that a high DP diet could inhibit the production of off-flavors.

Fish is not only a wonderful cuisine, but it also contributes essential nutrients for human health. In the study, fillet protein content was increased in the DP350 and DP400 groups. A similar pattern has been discovered in another investigation [52], which indicates that an optimal level of protein in the diet could improve the quality of fish fillets by raising fillet protein content. Food lipids not only offer energy but are also a vital source of critical fatty acids in animals [53]. The fatty acid contents of fish fillets are easily changed by the fatty acid composition of the diet or dietary oil source [28, 54]. In the study, the DL300 group (fish oil utilization) had the greatest contents of EPA, DHA, and ∑n-3, as well as the lowest value of TI. The findings indicated that it had a high nutritional value of fatty acids for human health [55]. Based on the aforementioned results of fillet quality indicators, especially for redness value, texture, EPA+DHA contents, and TI value, the minimum recommended DP and DL for adult triploid rainbow trout (≥3 kg) were 400 and 300 g kg^−1^, respectively.

## 5. Conclusion

In the study, for adult triploid rainbow trout (≥3 kg), the minimum recommended DP and DL based on growth performance were 400 and 250 g kg^−1^, respectively; DP and DL based on feed utilization were 350 and 200 g kg^−1^, respectively; DP and DL based on fillet quality were 400 and 300 g kg^−1^, respectively. In addition, a high DP diet (400 g kg^−1^) was beneficial to fish health, and a high DL diet (300 g kg^−1^) showed no influence on hepatic health.

## Figures and Tables

**Table 1:** The experimental diets' formulation and proximate components (g kg^−1^).

Ingredients	Diets (protein/lipid)								
	DP300/DL200	DP300/DL250	DP300/DL300	DP350/DL200	DP350/DL250	DP350/DL300	DP400/DL200	DP400/DL250	DP400/DL300
Brown fish meal^1^	370	370	370	440	440	440	510	510	510
Wheat meal^1^	150.5	150.5	150.5	150.5	150.5	150.5	150.5	150.5	150.5
Cassava starch^1^	150	150	150	100	100	100	50	50	50
Cellulose	138.6	88.6	38.6	126.6	76.6	26.6	113.6	63.6	13.6
Fish oil	133	183	233	125	175	225	118	168	218
Soybean oil	30	30	30	30	30	30	30	30	30
Vitamin-mineral premix^2^	10	10	10	10	10	10	10	10	10
Ca (H_2_PO_4_)_2_	8	8	8	8	8	8	8	8	8
Choline chloride	3	3	3	3	3	3	3	3	3
Calcium propionate	1	1	1	1	1	1	1	1	1
Ethoxyquin	0.5	0.5	0.5	0.5	0.5	0.5	0.5	0.5	0.5
Betaine	5	5	5	5	5	5	5	5	5
Astaxanthin^3^	0.4	0.4	0.4	0.4	0.4	0.4	0.4	0.4	0.4
Proximate analysis (*n* = 3)									
Moisture (g kg^−1^ diet)	56	40	42	50	48	47	53	48	49
Crude protein (g kg^−1^ diet, dry basis)	303	300	296	358	354	347	406	397	399
Crude lipid (g kg^−1^ diet, dry basis)	195	252	290	205	257	298	198	261	305
Gross energy (KJ g^−1^ diet)	20.9	23.0	24.3	21.5	23.4	24.8	21.3	23.5	25.2
P/E ratio (mg protein KJ^−1^)	14.5	13.1	12.2	16.7	15.2	14.0	19.1	16.9	15.8

^1^Brown fish meal containing 671 g kg^−1^ crude protein and 85 g kg^−1^ crude lipid. Wheat meal containing 165 g kg^−1^ crude protein and 26 g kg^−1^ crude lipid. Cassava starch containing 8.3 crude protein g kg^−1^ and 0.8 g kg^−1^ crude lipid. ^2^Vitamin-mineral premix included 5 g kg^−1^ vitamin premix and 5 g kg^−1^ mineral premix on the basis of our previous study [21]. ^3^Astaxanthin: 100 g kg^−1^.

**Table 2:** Growth performance and feed utilization of adult triploid rainbow trout in different diet groups (mean ± standard error).

Groups	Dietary protein/lipid levels (g kg^−1^)	Initial weight (kg)	Final weight (kg)	Survival (%)	Weight gain rate (%)	Feed conversion ratio	Feed intake (% day^−1^)
DP300DL200	300/200	3.32 ± 0.10	4.21 ± 0.01	98.7 ± 0.5	27.2 ± 3.7	1.81 ± 0.21	0.45 ± 0.05
DP300DL250	300/250	3.28 ± 0.07	4.27 ± 0.20	98.9 ± 0.3	29.8 ± 3.5	1.71 ± 0.28	0.53 ± 0.02
DP300DL300	300/300	3.22 ± 0.04	4.32 ± 0.10	98.7 ± 0.3	34.0 ± 2.0	1.53 ± 0.01	0.49 ± 0.03
DP350DL200	350/200	3.22 ± 0.04	4.24 ± 0.04	98.4 ± 0.3	32.1 ± 1.2	1.53 ± 0.10	0.51 ± 0.03
DP350DL250	350/250	3.27 ± 0.08	4.33 ± 0.18	98.3 ± 0.3	35.4 ± 2.6	1.31 ± 0.01	0.54 ± 0.03
DP350DL300	350/300	3.31 ± 0.05	4.47 ± 0.02	98.6 ± 0.3	35.2 ± 2.7	1.35 ± 0.06	0.48 ± 0.05
DP400DL200	400/200	3.16 ± 0.04	4.21 ± 0.01	98.9 ± 0.2	33.0 ± 1.5	1.38 ± 0.09	0.50 ± 0.03
DP400DL250	400/250	3.19 ± 0.01	4.43 ± 0.08	98.4 ± 0.2	38.6 ± 2.0	1.40 ± 0.07	0.55 ± 0.01
DP400DL300	400/300	3.21 ± 0.03	4.46 ± 0.01	98.0 ± 0.5	39.1 ± 1.4	1.23 ± 0.08	0.54 ± 0.04

Protein levels	300	3.27 ± 0.04	4.27 ± 0.07	98.8 ± 0.2	30.3 ± 1.9^A^	1.68 ± 0.11^B^	0.49 ± 0.02
	350	3.26 ± 0.03	4.35 ± 0.05	98.5 ± 0.1	34.2 ± 1.3^AB^	1.40 ± 0.05^A^	0.51 ± 0.02
	400	3.19 ± 0.02	4.36 ± 0.05	98.4 ± 0.2	36.9 ± 1.3^B^	1.34 ± 0.05^A^	0.53 ± 0.02

Lipid levels	200	3.23 ± 0.04	4.22 ± 0.01^X^	98.7 ± 0.2	30.7 ± 1.5^X^	1.57 ± 0.10	0.49 ± 0.02
	250	3.25 ± 0.03	4.34 ± 0.08^XY^	98.5 ± 0.2	34.6 ± 1.9^XY^	1.47 ± 0.10	0.54 ± 0.01
	300	3.25 ± 0.03	4.42 ± 0.04^Y^	98.4 ± 0.2	36.1 ± 1.3^Y^	1.37 ± 0.05	0.50 ± 0.02

Protein levels		0.158	0.377	0.331	0.017	0.014	0.391
Lipid levels		0.886	0.047	0.626	0.048	0.221	0.119
Protein levels × lipid levels		0.425	0.848	0.403	0.863	0.914	0.788

Data in the same column with different superscripts (A, B, and C based on two-way ANOVA) represent that the significant difference is discovered in the dietary protein level (*P* < 0.05), and those with different superscripts (X, Y, and Z based on two-way ANOVA) represent that the significant difference is discovered in the dietary lipid level (*P* < 0.05).

**Table 3:** Body indices of adult triploid rainbow trout in different diet groups (mean ± standard error).

Groups	Dietary protein/lipid levels (g kg^−1^)	Condition factor (CF)	Viscerosomatic index (VSI, %)	Hepatosomatic index (HSI, %)	Abdominal fat index (AFI)	Relative intestine length (RIL)	Fillet yield (FY, %)
DP300DL200	300/200	2.13 ± 0.07	13.8 ± 0.4	1.35 ± 0.02^ab^	0.084 ± 0.004	0.42 ± 0.01^a^	62.5 ± 0.2
DP300DL250	300/250	2.08 ± 0.05	13.5 ± 0.3	1.31 ± 0.02^ab^	0.083 ± 0.002	0.48 ± 0.01^c^	62.2 ± 0.5
DP300DL300	300/300	2.20 ± 0.06	15.1 ± 0.7	1.38 ± 0.02^b^	0.092 ± 0.006	0.45 ± 0.01^b^	61.5 ± 0.3
DP350DL200	350/200	2.21 ± 0.06	14.7 ± 0.2	1.35 ± 0.04^ab^	0.097 ± 0.001	0.46 ± 0.01^bc^	62.0 ± 0.1
DP350DL250	350/250	2.17 ± 0.05	14.8 ± 0.3	1.29 ± 0.02^ab^	0.095 ± 0.004	0.47 ± 0.01^bc^	61.9 ± 0.5
DP350DL300	350/300	2.07 ± 0.14	15.7 ± 0.7	1.52 ± 0.04^c^	0.099 ± 0.006	0.47 ± 0.01^bc^	61.7 ± 0.4
DP400DL200	400/200	2.16 ± 0.06	13.3 ± 0.1	1.25 ± 0.02^a^	0.083 ± 0.002	0.44 ± 0.00^ab^	63.7 ± 0.2
DP400DL250	400/250	2.17 ± 0.06	13.7 ± 0.4	1.33 ± 0.05^ab^	0.081 ± 0.003	0.45 ± 0.02^ab^	63.2 ± 0.3
DP400DL300	400/300	2.17 ± 0.07	15.2 ± 0.2	1.32 ± 0.03^ab^	0.096 ± 0.003	0.45 ± 0.01^ab^	62.0 ± 0.2

Protein levels	300	2.14 ± 0.04	14.1 ± 0.3^A^	1.35 ± 0.01^AB^	0.086 ± 0.003^A^	0.45 ± 0.01^A^	62.1 ± 0.2^A^
	350	2.15 ± 0.05	15.1 ± 0.3^B^	1.39 ± 0.03^B^	0.097 ± 0.002^B^	0.47 ± 0.01^B^	61.9 ± 0.2^A^
	400	2.17 ± 0.04	14.1 ± 0.3^A^	1.30 ± 0.02^A^	0.087 ± 0.002^A^	0.45 ± 0.01^A^	63.0 ± 0.2^B^

Lipid levels	200	2.16 ± 0.04	13.9 ± 0.2^X^	1.32 ± 0.02^X^	0.088 ± 0.002^X^	0.44 ± 0.01^X^	62.7 ± 0.2^Y^
	250	2.14 ± 0.03	14.0 ± 0.2^X^	1.31 ± 0.02^X^	0.086 ± 0.002^X^	0.47 ± 0.01^Y^	62.4 ± 0.3^Y^
	300	2.15 ± 0.05	15.3 ± 0.3^Y^	1.41 ± 0.03^Y^	0.096 ± 0.003^Y^	0.46 ± 0.00^XY^	61.7 ± 0.2^X^

Protein levels		0.892	0.005	0.006	0.002	0.046	≤0.001
Lipid levels		0.934	≤0.001	≤0.001	0.011	0.009	≤0.001
Protein levels × lipid levels		0.537	0.669	0.004	0.688	0.028	0.335

Data in the same column with different superscripts (a, b,…based on one-way ANOVA) represent that the significant difference is discovered in the interaction (*P* < 0.05), those with different superscripts (A, B, and C based on two-way ANOVA) represent that the significant difference is discovered in the dietary protein level (*P* < 0.05), and those with different superscripts (X, Y, and Z based on two-way ANOVA) represent that the significant difference is discovered in the dietary lipid level (*P* < 0.05).

**Table 4:** Liver and fillet tissue compositions of adult triploid rainbow trout in different diet groups (mean ± standard error).

Groups	Dietary protein/lipid levels (g kg^−1^)	Liver tissue compositions			Fillet tissue compositions	
		Crude lipid (mg g^−1^ liver tissue)	Crude protein (mg g^−1^ liver tissue)	Liver glycogen (mg g^−1^ liver tissue)	Crude lipid (mg g^−1^ fillet tissue)	Crude protein (mg g^−1^ fillet tissue)
DP300DL200	300/200	69.4 ± 3.0	159 ± 3	29.5 ± 1.6	140 ± 7	205 ± 1
DP300DL250	300/250	67.5 ± 4.8	157 ± 3	39.7 ± 2.3	127 ± 6	207 ± 2
DP300DL300	300/300	69.5 ± 3.7	156 ± 2	33.5 ± 2.2	143 ± 8	203 ± 2
DP350DL200	350/200	71.9 ± 6.0	159 ± 2	38.1 ± 3.9	135 ± 4	208 ± 1
DP350DL250	350/250	71.0 ± 7.6	159 ± 2	33.9 ± 3.5	142 ± 4	207 ± 1
DP350DL300	350/300	67.8 ± 4.8	154 ± 3	47.5 ± 7.5	136 ± 7	210 ± 2
DP400DL200	400/200	90.6 ± 10.1	170 ± 5	22.7 ± 3.8	121 ± 9	212 ± 2
DP400DL250	400/250	86.5 ± 5.9	162 ± 2	24.2 ± 1.6	129 ± 5	209 ± 2
DP400DL300	400/300	74.9 ± 2.4	171 ± 3	33.1 ± 3.6	137 ± 4	206 ± 1

Protein levels	300	68.8 ± 2.1^A^	157 ± 2^A^	34.2 ± 1.8^B^	136 ± 4	205 ± 1^A^
	350	70.2 ± 3.3^A^	157 ± 1^A^	39.8 ± 3.3^B^	137 ± 3	208 ± 1^B^
	400	84.0 ± 4.1^B^	168 ± 2^B^	26.7 ± 2.3^A^	129 ± 4	209 ± 1^B^

Lipid levels	200	77.3 ± 4.6	163 ± 2	30.1 ± 2.8^X^	132 ± 4	209 ± 1
	250	75.0 ± 4.1	160 ± 1	32.6 ± 2.6^X^	132 ± 3	208 ± 1
	300	70.7 ± 2.2	160 ± 3	38.0 ± 3.5^Y^	139 ± 4	206 ± 1

Protein levels		0.006	≤0.001	0.002	0.219	0.018
Lipid levels		0.385	0.485	0.049	0.370	0.231
Protein levels × lipid levels		0.679	0.231	0.133	0.250	0.150

Data in the same column with different superscripts (A, B, and C based on two-way ANOVA) represent that the significant difference is discovered in the dietary protein level (*P* < 0.05), and those with different superscripts (X, Y, and Z based on two-way ANOVA) represent that the significant difference is discovered in the dietary lipid level (*P* < 0.05).

**Table 5:** Plasma indexes and metabolism enzyme activities of adult triploid rainbow trout in different diet groups (mean ± standard error).

Groups	Dietary protein/lipid levels (g kg^−1^)	Plasma indexes		Amino acid metabolism enzymes in liver			Lipid metabolism enzymes in liver		
		Alanine aminotransferase (U L^−1^)	Aspartate aminotransferase (U L^−1^)	Glutamate dehydrogenase (U mgprot^−1^)	Alanine aminotransferase (U gprot^−1^)	Aspartate aminotransferase (U gprot^−1^)	Hepatic lipase (U mgprot^−1^)	Lipoprotein lipase (U mgprot^−1^)	Total lipase (U mgprot^−1^)
DP300DL200	300/200	5.33 ± 0.33	368 ± 64	15.02 ± 2.52^d^	12.88 ± 0.90^bc^	22.7 ± 1.5^abc^	1.54 ± 0.44^b^	0.30 ± 0.03^a^	1.83 ± 0.41^b^
DP300DL250	300/250	6.33 ± 1.45	427 ± 83	4.93 ± 0.44^a^	12.53 ± 1.06^bc^	25.0 ± 2.2^cd^	0.70 ± 0.11^a^	0.27 ± 0.01^a^	0.97 ± 0.11^a^
DP300DL300	300/300	3.00 ± 1.00	212 ± 55	12.17 ± 1.16^cd^	13.02 ± 0.69^c^	31.4 ± 2.2^c^	0.82 ± 0.11^a^	0.51 ± 0.06^b^	1.33 ± 0.13^ab^
DP350DL200	350/200	3.67 ± 0.67	252 ± 36	13.74 ± 1.98^d^	8.45 ± 0.69^a^	16.9 ± 1.7^a^	0.64 ± 0.08^a^	0.34 ± 0.01^a^	0.98 ± 0.10^a^
DP350DL250	350/250	5.33 ± 0.33	303 ± 26	13.29 ± 2.41^d^	11.26 ± 1.12^abc^	21.4 ± 1.8^abc^	0.60 ± 0.04^a^	0.35 ± 0.01^a^	0.95 ± 0.03^a^
DP350DL300	350/300	5.33 ± 0.88	287 ± 15	6.76 ± 1.48^ab^	12.33 ± 0.65^bc^	23.0 ± 1.0^abc^	0.99 ± 0.04^a^	0.77 ± 0.09^c^	1.76 ± 0.12^b^
DP400DL200	400/200	4.67 ± 0.33	217 ± 23	5.21 ± 0.44^a^	13.55 ± 1.87^c^	24.7 ± 4.6^bcd^	0.87 ± 0.06^a^	0.52 ± 0.07^b^	1.40 ± 0.11^ab^
DP400DL250	400/250	8.67 ± 3.18	453 ± 132	7.94 ± 0.65^abc^	11.54 ± 1.69^abc^	26.7 ± 2.8^cd^	0.73 ± 0.06^a^	0.31 ± 0.01^a^	1.04 ± 0.06^a^
DP400DL300	400/300	7.00 ± 0.00	397 ± 22	10.97 ± 2.02^bcd^	9.34 ± 0.42^ab^	17.3 ± 1.0^ab^	0.55 ± 0.02^a^	0.34 ± 0.03^a^	0.89 ± 0.03^a^

Protein levels	300	4.89 ± 0.72	336 ± 47	10.71 ± 1.54	12.81 ± 0.47	26.4 ± 1.5^B^	1.02 ± 0.18	0.36 ± 0.04^A^	1.38 ± 0.17
	350	4.78 ± 0.43	281 ± 15	11.27 ± 1.42	10.68 ± 0.66	20.4 ± 1.1^A^	0.74 ± 0.06	0.49 ± 0.07^B^	1.23 ± 0.12
	400	6.78 ± 1.09	356 ± 53	8.04 ± 0.97	11.48 ± 0.93	22.9 ± 2.1^AB^	0.72 ± 0.05	0.39 ± 0.04^A^	1.11 ± 0.08

Lipid levels	200	4.56 ± 0.34	279 ± 32	11.32 ± 1.64	11.63 ± 0.95	21.5 ± 1.9	1.02 ± 0.18^Y^	0.39 ± 0.04^X^	1.40 ± 0.17^Y^
	250	6.78 ± 1.13	394 ± 51	8.72 ± 1.29	11.78 ± 0.71	24.4 ± 1.4	0.68 ± 0.04^X^	0.31 ± 0.01^X^	0.99 ± 0.04^X^
	300	5.11 ± 0.70	299 ± 32	9.97 ± 1.09	11.57 ± 0.57	23.9 ± 1.9	0.78 ± 0.06^XY^	0.54 ± 0.06^Y^	1.33 ± 0.12^XY^

Protein levels		0.128	0.333	0.053	0.078	0.015	0.054	0.005	0.143
Lipid levels		0.115	0.077	0.175	0.972	0.279	0.044	≤0.001	0.009
Protein levels × lipid levels		0.353	0.112	≤0.001	0.021	0.008	0.019	≤0.001	≤0.001

Data in the same column with different superscripts (a, b,…based on one-way ANOVA) represent that the significant difference is discovered in the interaction (*P* < 0.05), those with different superscripts (A, B, and C based on two-way ANOVA) represent that the significant difference is discovered in the dietary protein level (*P* < 0.05), and those with different superscripts (X, Y, and Z based on two-way ANOVA) represent that the significant difference is discovered in the dietary lipid level (*P* < 0.05).

**Table 6:** Fillet color of adult triploid rainbow trout in different diet groups (mean ± standard error).

Groups	Dietary protein/lipid levels (g kg^−1^)	Fillet redness value	Lightness (*L*^∗^)	Redness (*a*^∗^)	Yellowness (*b*^∗^)	Chroma (*C*^∗^_ab_)	Hue (*H*_ab_°)
DP300DL200	300/200	29.6 ± 0.2	46.1 ± 1.4	20.2 ± 0.5^cd^	24.4 ± 0.5	31.9 ± 0.5^cd^	50.8 ± 1.0
DP300DL250	300/250	29.8 ± 0.2	45.7 ± 1.3	19.8 ± 0.6^bcd^	23.8 ± 0.9	30.9 ± 1.0^bcd^	49.4 ± 1.3
DP300DL300	300/300	29.7 ± 0.2	46.7 ± 1.6	19.4 ± 0.4^bc^	23.9 ± 0.5	30.8 ± 0.5^bcd^	51.7 ± 1.0
DP350DL200	350/200	29.9 ± 0.2	44.9 ± 1.4	19.3 ± 0.4^bc^	22.1 ± 0.6	29.3 ± 0.7^ab^	49.2 ± 0.9
DP350DL250	350/250	29.6 ± 0.2	45.8 ± 1.5	18.3 ± 0.6^ab^	22.1 ± 0.8	28.6 ± 1.0^ab^	50.8 ± 0.7
DP350DL300	350/300	29.9 ± 0.2	45.9 ± 1.4	17.1 ± 0.4^a^	22.0 ± 0.7	27.9 ± 0.9^a^	51.8 ± 0.5
DP400DL200	400/200	30.0 ± 0.2	44.7 ± 1.2	18.4 ± 0.6^ab^	22.9 ± 0.8	29.4 ± 1.2^abc^	51.6 ± 0.7
DP400DL250	400/250	29.8 ± 0.2	45.7 ± 1.8	19.8 ± 0.5^bcd^	24.6 ± 0.5	32.0 ± 0.7^d^	50.9 ± 0.8
DP400DL300	400/300	29.9 ± 0.2	44.8 ± 1.9	21.3 ± 0.6^d^	24.5 ± 0.4	33.1 ± 0.6^d^	49.2 ± 1.0

Protein levels	300	29.7 ± 0.1	46.1 ± 0.8	19.8 ± 0.3^B^	24.1 ± 0.4^B^	31.2 ± 0.4^B^	50.6 ± 0.6
	350	29.8 ± 0.1	45.5 ± 0.8	18.2 ± 0.3^A^	22.0 ± 0.4^A^	28.6 ± 0.5^A^	50.6 ± 0.5
	400	29.9 ± 0.1	45.1 ± 0.9	19.9 ± 0.4^B^	24.0 ± 0.4^B^	31.5 ± 0.5^B^	50.6 ± 0.5

Lipid levels	200	29.8 ± 0.1	45.2 ± 0.8	19.3 ± 0.3	23.1 ± 0.4	30.2 ± 0.5	50.5 ± 0.5
	250	29.7 ± 0.1	45.7 ± 0.9	19.3 ± 0.3	23.5 ± 0.5	30.5 ± 0.6	50.4 ± 0.6
	300	29.8 ± 0.1	45.8 ± 0.9	19.3 ± 0.4	23.5 ± 0.3	30.6 ± 0.5	50.9 ± 0.5

Protein levels		0.478	0.675	≤0.001	≤0.001	≤0.001	0.998
Lipid levels		0.781	0.877	0.991	0.749	0.846	0.764
Protein levels × lipid levels		0.738	0.973	≤0.001	0.389	0.016	0.085

Data in the same column with different superscripts (a, b,...based on one-way ANOVA) represent that the significant difference is discovered in the interaction (*P* < 0.05), those with different superscripts (A, B, and C based on two-way ANOVA) represent that the significant difference is discovered in the dietary protein level (*P* < 0.05).

**Table 7:** Fillet texture of adult triploid rainbow trout in different diet groups (mean ± standard error).

Groups	Dietary protein/lipid levels (g kg^−1^)	Fracture (*N*)	Hardness (*N*)	Adhesiveness (mJ)	Springiness (mm)	Chewiness (mJ)	Cohesiveness	Water holding capacity (%)	pH
DP300DL200	300/200	6.61 ± 0.29	7.66 ± 0.21	4.67 ± 0.43	9.47 ± 0.47^d^	14.21 ± 1.43^c^	0.21 ± 0.02	90.7 ± 0.6^bcd^	6.13 ± 0.02
DP300DL250	300/250	6.04 ± 0.38	6.85 ± 0.33	3.61 ± 0.55	7.02 ± 0.48^c^	10.30 ± 0.79^ab^	0.23 ± 0.03	90.2 ± 0.3^abc^	6.18 ± 0.03
DP300DL300	300/300	6.56 ± 0.48	7.74 ± 0.26	4.72 ± 0.47	7.37 ± 0.50^c^	10.37 ± 0.76^ab^	0.20 ± 0.01	88.5 ± 0.5^a^	6.18 ± 0.02
DP350DL200	350/200	7.58 ± 0.23	8.56 ± 0.40	7.70 ± 0.29	4.97 ± 0.21^a^	7.59 ± 0.56^a^	0.16 ± 0.01	89.3 ± 0.4^ab^	6.17 ± 0.02
DP350DL250	350/250	6.41 ± 0.27	7.85 ± 0.34	6.67 ± 0.22	5.13 ± 0.28^ab^	9.13 ± 0.90^ab^	0.21 ± 0.02	92.2 ± 0.8^d^	6.17 ± 0.01
DP350DL300	350/300	6.17 ± 0.25	8.25 ± 0.41	6.25 ± 0.44	6.27 ± 0.29^bc^	10.66 ± 1.22^b^	0.20 ± 0.02	90.3 ± 0.3^abcd^	6.16 ± 0.03
DP400DL200	400/200	7.21 ± 0.24	8.75 ± 0.25	5.90 ± 0.37	6.29 ± 0.64^bc^	9.36 ± 1.01^ab^	0.17 ± 0.01	91.2 ± 0.8^bcd^	6.19 ± 0.02
DP400DL250	400/250	6.07 ± 0.43	7.47 ± 0.39	4.96 ± 0.23	7.15 ± 0.44^c^	10.12 ± 0.67^ab^	0.20 ± 0.03	90.8 ± 0.8^bcd^	6.18 ± 0.02
DP400DL300	400/300	5.79 ± 0.41	7.40 ± 0.41	4.94 ± 0.35	6.69 ± 0.36^c^	8.85 ± 0.78^ab^	0.19 ± 0.02	91.3 ± 0.7^cd^	6.16 ± 0.02

Protein levels	300	6.40 ± 0.22	7.42 ± 0.17^A^	4.34 ± 0.29^A^	7.95 ± 0.33^C^	11.63 ± 0.66^B^	0.21 ± 0.01	89.8 ± 0.4^A^	6.16 ± 0.02
	350	6.72 ± 0.19	8.22 ± 0.22^B^	6.87 ± 0.21^C^	5.45 ± 0.18^A^	9.13 ± 0.56^A^	0.19 ± 0.01	90.6 ± 0.4^AB^	6.17 ± 0.01
	400	6.36 ± 0.24	7.87 ± 0.23^AB^	5.26 ± 0.20^B^	6.71 ± 0.28^B^	9.45 ± 0.47^A^	0.19 ± 0.01	91.1 ± 0.4^B^	6.18 ± 0.01

Lipid levels	200	7.13 ± 0.16^Y^	8.32 ± 0.18^Y^	6.09 ± 0.30^Y^	6.91 ± 0.42	10.39 ± 0.76	0.18 ± 0.01	90.4 ± 0.4	6.16 ± 0.01
	250	6.17 ± 0.21^X^	7.39 ± 0.21^X^	5.08 ± 0.30^X^	6.43 ± 0.28	9.85 ± 0.45	0.21 ± 0.01	91.1 ± 0.4	6.18 ± 0.01
	300	6.17 ± 0.23^X^	7.79 ± 0.21^XY^	5.31 ± 0.26^XY^	6.78 ± 0.23	9.96 ± 0.54	0.20 ± 0.01	90.0 ± 0.4	6.17 ± 0.01

Protein levels		0.377	0.019	≤0.001	≤0.001	0.003	0.149	0.038	0.667
Lipid levels		≤0.001	0.005	0.005	0.372	0.758	0.101	0.111	0.773
Protein levels × lipid levels		0.275	0.302	0.257	≤0.001	0.003	0.644	0.009	0.553

Data in the same column with different superscripts (a, b,…based on one-way ANOVA) represent that the significant difference is discovered in the interaction (*P* < 0.05), those with different superscripts (A, B, and C based on two-way ANOVA) represent that the significant difference is discovered in the dietary protein level (*P* < 0.05), and those with different superscripts (X, Y, and Z based on two-way ANOVA) represent that the significant difference is discovered in the dietary lipid level (*P* < 0.05).

**Table 8:** Fillet odor of adult triploid rainbow trout in different diet groups (mean ± standard error).

Volatile compounds	Ng g^−1^ muscle									Two-way ANOVA (*P* < 0.05)		
	DP300DL200	DP300DL250	DP300DL300	DP350DL200	DP350DL250	DP350DL300	DP400DL200	DP400DL250	DP400DL300	Protein levels	Lipid levels	Protein × lipid
1-Heptanol	26.0 ± 1.5	31.8 ± 5.0	27.7 ± 5.1	42.0 ± 4.6	56.5 ± 4.9	53.2 ± 4.5	35.6 ± 8.8	43.6 ± 11.6	51.5 ± 9.9	0.013	0.120	0.826
1-Octen-3-ol	93 ± 7	138 ± 24	106 ± 25	142 ± 22	213 ± 28	205 ± 13	89 ± 25	130 ± 40	181 ± 40	0.024	0.014	0.454
2,3-Pentanedione	27.4 ± 3.4	33.7 ± 7.9	24.4 ± 3.8	31.8 ± 4.2	53.3 ± 5.4	32.3 ± 4.7	25.4 ± 5.9	22.3 ± 8.3	29.8 ± 7.5	0.025	0.070	0.161
2,3-Octanedione	39.4 ± 4.4	39.8 ± 6.0	42.8 ± 7.4	56.4 ± 4.5	63.0 ± 4.7	50.9 ± 7.9	52.4 ± 11.3	44.5 ± 9.6	47.9 ± 9.3	0.126	0.894	0.788
3,5-Octadien-2-one	96 ± 8^a^	163 ± 27^ab^	126 ± 22^ab^	115 ± 15^ab^	202 ± 21^bc^	278 ± 14^c^	100 ± 31^a^	168 ± 39^ab^	275 ± 56^c^	0.095	≤0.001	0.037
Hexanal	280 ± 25	428 ± 94	298 ± 73	428 ± 67	677 ± 123	510 ± 35	253 ± 65	312 ± 90	410 ± 88	0.008	0.029	0.363
(E)-2-Hexenal	16.4 ± 1.9	20.0 ± 3.0	17.4 ± 2.9	20.6 ± 2.8	29.3 ± 1.7	30.2 ± 4.2	14.1 ± 2.6	17.2 ± 3.4	23.0 ± 4.5	0.003	0.029	0.496
Heptanal	42.2 ± 5.2	61.0 ± 11.6	44.2 ± 7.7	68.2 ± 7.3	78.4 ± 7.4	82.8 ± 20.0	49.1 ± 10.1	55.8 ± 13.3	78.7 ± 20.3	0.115	0.141	0.575
(E)-2-Heptenal	14.1 ± 3.0	23.8 ± 6.4	24.6 ± 7.1	28.7 ± 5.2	46.9 ± 3.3	39.1 ± 6.9	20.9 ± 5.3	16.5 ± 5.3	26.0 ± 6.6	≤0.001	0.108	0.336
Octanal	63.4 ± 4.9	68.1 ± 10.2	71.0 ± 12.6	98.5 ± 9.4	127.9 ± 8.1	113.7 ± 10.7	81.7 ± 17.4	86.8 ± 18.4	93.7 ± 17.3	0.003	0.257	0.924
(*E, E*)-2,4-Heptadienal	17.7 ± 2.3	19.2 ± 3.1	30.6 ± 4.7	25.1 ± 3.1	34.0 ± 1.9	33.6 ± 3.7	27.5 ± 4.2	28.2 ± 5.0	34.8 ± 6.7	0.124	0.032	0.816
(E)-2-Octenal	18.9 ± 2.8	32.0 ± 7.8	21.2 ± 3.8	27.1 ± 3.0	40.9 ± 6.5	38.0 ± 4.6	21.3 ± 4.0	22.6 ± 4.1	35.6 ± 8.4	0.136	0.022	0.119
Nonanal	94 ± 10	118 ± 21	100 ± 20	138 ± 13	161 ± 18	154 ± 16	141 ± 26	135 ± 21	136 ± 28	0.156	0.381	0.887
(*E, Z*)-2,6-Nonadienal	17.2 ± 1.8	22.5 ± 3.1	21.2 ± 3.1	22.7 ± 2.7	35.1 ± 2.2	38.5 ± 3.9	18.4 ± 3.6	25.4 ± 5.0	35.7 ± 6.9	0.017	≤0.001	0.412
(E)-2-Nonenal	9.9 ± 0.8	12.7 ± 2.3	13.5 ± 3.0	14.6 ± 2.3	18.8 ± 3.0	19.6 ± 2.5	12.3 ± 2.5	14.7 ± 3.0	18.3 ± 3.7	0.170	0.058	0.955
Decanal	8.9 ± 0.6	11.5 ± 2.1	12.8 ± 2.5	14.7 ± 1.4	19.0 ± 1.7	18.8 ± 2.0	16.1 ± 3.1	16.3 ± 3.1	16.8 ± 3.4	0.041	0.154	0.918
(*E, E*)-2,4-Nonadienal	3.41 ± 0.45	5.99 ± 1.67	3.69 ± 0.78	4.21 ± 0.73	5.41 ± 1.02	5.08 ± 0.70	3.66 ± 0.93	4.54 ± 0.89	7.20 ± 1.60	0.813	0.076	0.190
(E)-2-Decenal	7.06 ± 0.52	8.30 ± 1.86	9.98 ± 2.17	11.12 ± 0.96	15.19 ± 2.70	11.85 ± 1.12	11.40 ± 2.93	10.17 ± 1.41	11.54 ± 2.47	0.148	0.378	0.673
Undecanal	8.9 ± 1.0	12.8 ± 2.6	16.6 ± 4.3	17.5 ± 1.8	19.2 ± 1.9	18.1 ± 3.4	22.8 ± 3.8	21.4 ± 3.5	22.7 ± 4.3	0.025	0.361	0.679
2-Ethyl-furan	6.80 ± 0.50	10.50 ± 2.61	8.89 ± 1.65	7.93 ± 1.27	13.42 ± 1.00	13.31 ± 1.08	7.69 ± 1.97	10.31 ± 2.30	11.62 ± 2.24	0.076	0.010	0.922
Total	890 ± 66	1548 ± 267	1089 ± 212	1319 ± 178	1908 ± 224	1722 ± 139	973 ± 253	1186 ± 278	1547 ± 315	0.027	0.027	0.500
n-3 derived^1^	154 ± 14	236 ± 34	208 ± 32	191 ± 21	313 ± 25	394 ± 24	163 ± 47	249 ± 53	380 ± 75	0.066	≤0.001	0.136
n-6 derived^1^	458 ± 39	696 ± 142	507 ± 117	708 ± 103	1074 ± 166	879 ± 73	445 ± 108	552 ± 152	750 ± 162	0.011	0.027	0.384
n-9 derived^1^	241 ± 20	298 ± 50	265 ± 49	372 ± 32	457 ± 41	429 ± 52	334 ± 67	348 ± 66	388 ± 79	0.039	0.228	0.901

Data in the same row with different superscripts (a, b,…based on one-way ANOVA) represent that a significant difference is discovered in the interaction (*P* < 0.05). ^1^n-3 derived, n-6 derived, and n-9 derived represent subtotal concentration of compounds derived by n-3 fatty acids, n-6 fatty acids, and n-9 fatty acids, respectively [23].

**Table 9:** Fillet fatty acid compositions of adult triploid rainbow trout in different diet groups (mean ± standard error).

Groups	g kg^−1^ muscle (wet basis)									Two-way ANOVA (*P* < 0.05)		
	DP300DL200	DP300DL250	DP300DL300	DP350DL200	DP350DL250	DP350DL300	DP400DL200	DP400DL250	DP400DL300	Protein levels	Lipid levels	Protein × lipid
C18:2n-6	15.8 ± 1.1	14.7 ± 0.9	14.9 ± 0.7	14.5 ± 0.6	14.2 ± 0.6	12.9 ± 0.8	14.2 ± 1.1	13.7 ± 0.7	14.5 ± 0.5	0.119	0.437	0.675
C18:3n-3	0.17 ± 0.02	0.14 ± 0.01	0.15 ± 0.01	0.15 ± 0.01	0.13 ± 0.01	0.13 ± 0.01	0.15 ± 0.02	0.14 ± 0.01	0.15 ± 0.01	0.439	0.222	0.899
C20:4n-6	0.36 ± 0.02	0.34 ± 0.02	0.38 ± 0.02	0.47 ± 0.16	0.34 ± 0.02	0.35 ± 0.02	0.32 ± 0.02	0.35 ± 0.02	0.37 ± 0.01	0.708	0.657	0.583
C20:5n-3 (EPA)	2.02 ± 0.09	1.78 ± 0.10	2.22 ± 0.14	1.48 ± 0.15	1.85 ± 0.10	2.10 ± 0.14	1.72 ± 0.12	2.01 ± 0.08	2.18 ± 0.09	0.080	≤0.001	0.075
C22:6n-3 (DHA)	5.77 ± 0.23	5.12 ± 0.20	6.02 ± 0.35	4.73 ± 0.14	5.25 ± 0.26	5.70 ± 0.32	4.98 ± 0.29	5.67 ± 0.20	5.99 ± 0.19	0.118	≤0.001	0.080
TFA^1^	77.2 ± 4.5	68.0 ± 3.8	76.0 ± 4.1	68.5 ± 2.4	69.8 ± 2.8	68.0 ± 4.1	67.0 ± 5.1	71.0 ± 3.1	73.7 ± 2.4	0.258	0.614	0.355
SFA^1^	20.8 ± 1.0	18.1 ± 1.0	20.3 ± 1.2	18.3 ± 0.6	18.8 ± 0.8	18.3 ± 1.1	18.3 ± 1.4	19.5 ± 0.8	19.7 ± 0.7	0.281	0.711	0.294
MUFA^1^	31.2 ± 2.0	26.8 ± 1.7	31.0 ± 1.9	27.9 ± 1.2	28.3 ± 1.2	27.6 ± 1.9	26.4 ± 2.2	28.8 ± 1.4	29.9 ± 1.2	0.433	0.528	0.263
PUFA^1^	25.2 ± 1.5	23.1 ± 1.2	24.7 ± 1.1	22.3 ± 0.8	22.7 ± 0.9	22.1 ± 1.3	22.3 ± 1.5	22.8 ± 1.0	24.1 ± 0.7	0.104	0.690	0.599
∑n-3^1^	7.95 ± 0.31	7.03 ± 0.29	8.38 ± 0.49	6.35 ± 0.22	7.23 ± 0.36	7.93 ± 0.47	6.84 ± 0.41	7.81 ± 0.28	8.32 ± 0.27	0.086	≤0.001	0.068
∑n-6^1^	17.3 ± 1.2	16.0 ± 0.9	16.3 ± 0.8	15.9 ± 0.6	15.5 ± 0.6	14.2 ± 0.9	15.5 ± 1.1	14.9 ± 0.8	15.8 ± 0.5	0.125	0.437	0.681
AI^1^	0.48 ± 0.01	0.47 ± 0.01	0.48 ± 0.01	0.46 ± 0.01	0.48 ± 0.01	0.49 ± 0.01	0.48 ± 0.01	0.50 ± 0.01	0.49 ± 0.01	0.133	0.148	0.111
TI^1^	0.42 ± 0.01	0.41 ± 0.01	0.40 ± 0.01	0.43 ± 0.01	0.42 ± 0.01	0.40 ± 0.01	0.43 ± 0.01	0.42 ± 0.01	0.40 ± 0.01	0.568	≤0.001	0.439

^1^TFA, SFA, MUFA, PUFA, ∑n-3, ∑n-6, AI, and TI represent total fatty acids, saturated fatty acids, monounsaturated fatty acids, polyunsaturated fatty acids, n-3 polyunsaturated fatty acids, n-6 polyunsaturated fatty acids, atherogenicity index, and thrombogenicity index, respectively.

## Data Availability

The data that support the findings of this study are available from the corresponding author upon reasonable request.
